# Benefits of emergency department routine blood test performance on patients whose allocated triage category is not time critical: a retrospective study

**DOI:** 10.1186/s12913-024-11612-w

**Published:** 2024-10-17

**Authors:** Abdi D. Osman, Jocelyn Howell, Michael Yeoh, Damian Wilson, Virginia Plummer, George Braitberg

**Affiliations:** 1 Department of Critical Care, University of Melbourne, 157-159 Barry Street Parkville, Melbourne, VIC 3010 Australia; 2 Emergency Department, Austin Health, 145 Studley Rd, Heidelberg, Melbourne, VIC 3084 Australia; 3 College of Sports, Health and Engineering, Victoria University, University Blvd, St. Albans, Melbourne, VIC 3021 Australia; 4 Emergency Department, Sunshine Hospital, 176 Furlong Rd, St Albans, Melbourne, VIC 3021 Australia; 5 Institute of Health and Wellbeing, Federation University, 100 Clyde Rd, Berwick, Melbourne, VIC 3806 Australia

**Keywords:** Emergency department, Routine blood tests, Point of care test, Length of stay

## Abstract

**Introduction:**

Emergency department clinicians, and particularly nurses in triage, frequently perform routine blood tests on patients whose allocated triage category is not time critical (triage categories 3, 4 and 5 of the Australasian Triage Scale). Some observers have questioned the utility of routine blood testing in these acute healthcare settings given the cost and workload implications.

**Methods:**

A quantitative method using retrospective observational design was utilised guided by STROBE checklist. Electronic medical records of patient data collected at a quaternary Australian metropolitan hospital emergency department were reviewed.

**Results and discussion:**

A total of 74,878 adult patients attended the emergency department between 1st January and 31st December 2021 and a sample of 383 were randomly allocated for this study. Of the 383 patients included, 51% were female, age ranges were 18–99 years (mean 51.6). The majority were Australasian Triage scale (ATS) triage category 3 (55%) and 62% had blood tests performed. Blood test performance was found to be associated with advancing age (*p* < 0.001) but not with department occupancy as determined by the national emergency department overcrowding scale (*p* = 0.230).

**Conclusion:**

Blood testing in the emergency department in triaged non-time critical patients was found to be frequent thereby affecting nurses’ already stretched time resource. Older patients were found to be more likely to have a blood test. There is a positive correlation between blood test performance and length of stay in the emergency department.

## Introduction

Emergency department (ED) clinicians frequently undertake routine blood testing on adult patients. Blood tests are sometimes performed on first contact with the patient at triage, initiated by post-graduate trained nurse specialist staff prior to the patient being seen by a doctor. While the results may help with patient’s diagnosis and timely management, it is also costly to healthcare services, prolongs ED waiting times and raises ethical questions as it is invasive contrary to relevance on non-maleficence and justice [1, 2].

Australian EDs are guided by the Australasian College of Emergency Medicine (ACEM) and the Royal College of Pathologists of Australasia (RCPA) joint guideline on pathology testing in the ED [3]. There are diverse findings in the literature on the benefits or lack of benefits of performing routine blood tests on non-time critical ‘stable’ patients [4, 5]. There have also been national initiatives and campaigns such as, “*More is not always better when it comes to healthcare. A national conversation about reducing unnecessary tests*,* treatments and procedures*” by Choosing Wisely Australia [6] to ensure such tests are performed only when necessary.

This study will investigate the frequency and necessity of performing routine blood tests on patients in the ED who are triaged as lower acuity according to the Australasian Triage Scale (ATS), specifically triage categories 3, 4 and 5 [7]. Triage category 3 encompasses a broad spectrum of acuity, ranging from potentially life-threatening situations to urgent circumstances necessitating timely relief of discomfort or distress [7]. Therefore, the necessity of performing blood test for some individuals in this group cannot be ruled out.

The study’s primary aim was to evaluate whether association existed between blood test results and patient diagnosis or treatment. A secondary aim was to assess whether there was any association between blood testing and the busyness of the ED using the National Emergency Department Overcrowding Scale (NEDOCS) [8], patients advancing age and ED length of stay (EDLOS). The outcomes were expected to inform future interventions for safe reduction in the number of unnecessary tests.

## Method

### Study method and site

Quantitative method and retrospective design guided by STROBE checklist for observational studies [9] was used to review electronic medical records (EMR) for data collected at a major metropolitan teaching hospital of 670 beds with an annual ED census of around 90,000 patients situated in Victoria, Australia. The target population included triage categories 3, 4 and 5 adult patients who attended the ED between 1st January 2021 and 31st December 2021.

Ethics approval to conduct the study was obtained from the Institutional Office for research. Consent was waived given that, the data was de-identified and the study was observational in nature and participants are likely to have agreed to participate in this study.

### Sampling

A total of 74,878 adult patients attended the department during the study period, 14,383 patients were excluded due to their allocated triage categories which were higher than triage category 3 [7] and the rest were included. Using OpenEpi [10] with assumptions of 95% confidence interval, anticipated frequency of 50% (anticipated blood sampling frequency with an assumption that, all blood test affected diagnosis and treatment decision making), study power of 80% and design effect of 1, the resultant sample size was 383. Using SPSS™, the sample size was randomly selected through sampling from the included population and statistical analysis was undertaken.

### Data extraction

Variables extracted through report generation from the EMR of participants were patient demographics where age was categorised into six categories (18–25, 26–35, 36–45, 46–55, 56–65, > 65), triage category, date and time patient attended the department. This was followed by manual review on whether blood test was performed, and time performed, types of blood tests performed, time seen by the doctor, whether the patient had previous blood tests and last date. Discharge diagnosis was recorded as per International statistical Classification of Disease and related health problems (ICD) [11], length of stay in minutes and discharge destination.

### Retrospective chart review

The decision on whether the blood test results influenced management or diagnosis was determined by examination of the entries made in the patients discharge summary that clearly identified the consideration of blood tests in making a diagnosis and providing treatment. This was done by the lead researcher (a senior emergency nurse) with 25% of cases (*n* = 55) randomly selected (using SPSS™ version 27) for independent review by two of either senior ED physicians and/or a senior emergency nurse. A formal measure of inter-observer agreement was undertaken using kappa analysis.

### Analysis

Data was entered in a Microsoft Office Excel^®^ spreadsheet and imported to SPSS™ for analysis. If there were missing values, the data were presented as n (number of cases) / N (number of instances where the value was known) with no assumptions made about the missing data. Numerical data were presented in counts and percentages. Categorical data were analysed using chi-square tests or fisher’s exact tests as appropriate, ordinal and interval data with bivariate correlation and simple linear regression tests with statistical significance indicated by a two-sided *P* value < 0.05 and Confidence Interval (CI) range.

## Results

**Fig. 1:**
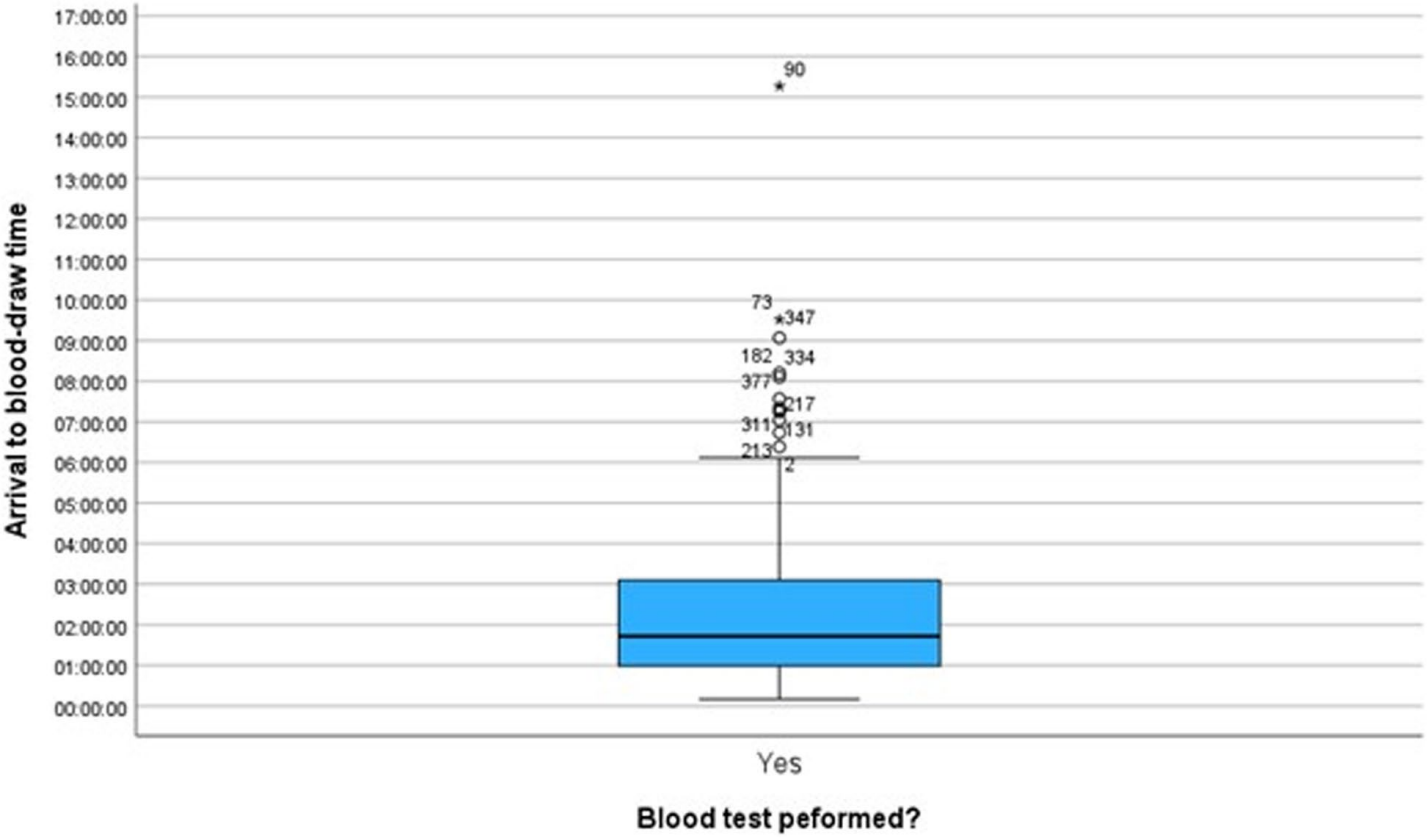
Arrival to blood test time

A sample of 383 cases were selected for analysis from a population of 60,495 eligible patient encounters. Of these, 51% were female and the age ranges were 18–99 years with a mean age of 51.6 years. The majority of the patients who presented were allocated triage category 3 (55%) followed by category 4 (37%). 62% of patients had blood tests performed, with an arrival to blood draw time range of 10 min to 9 h and 31 min with a single outlier for 15 h and 16 min and a mean time of 2 h 21 min (Fig. 1).

Full blood examination (FBE) and urea, electrolytes, and creatinine (UEC) were the most common tests with 98% of patients who had blood tests having FBE and UEC included, while 27% had the combination of FBE and UEC only. The most frequent tests accompanying the FBE and UEC were liver function test (LFT) at about 40%, troponin 27%, C-Reactive Protein (CRP) 21%, lipase 14% and d-dimer/coagulation test 13%.

**Table 1:** Association between increased blood testing and age, gender, EDLOS, acuity and NEDOCS (*n* = 766)

Variable	Blood test performed	Blood test not performed	*p* value
Advancing age (≥65 years) *n*=122	93 (76%)	29 (24%)	*p*<0.001
Mean EDLOS (Minutes)	437 (62%)	196 (38%)	*p*<0.001
Acuity (ATS)	Cat 3 = 312 (40.7%)	Cat 3 = 112 (14.6%)	*P*<0.001
	Cat 4 = 154 (20.1%)	Cat 4 =132 (17.2%)	
	Cat 5 =10 (1.3%)	Cat 5 = 46 (6%)	
Gender	Female = 246 (32.1%)	Female = 144 (18.8%)	(*p*=0.701
	Male = 230 (30%)	Male = 146 (19.1%)	
NEDOCS (National Emergency Department Overcrowding Scale) *n*=382	Busy = 21 (6.1%)	Busy = 12 (3.5%)	*p*=0.230
	Extremely busy =82 (24%)	Extremely busy =48 (14%)	
	Overcrowded = 81 (23.7%)	Overcrowded = 60 (17.5%)	
	Severely Overcrowded = 50 (14.6%)	Severely Overcrowded = 20 (5.8%)	
	Dangerously Overcrowded = 3 (0.9%)	Dangerously Overcrowded = 5 (1.5%)	

**Fig. 2:**
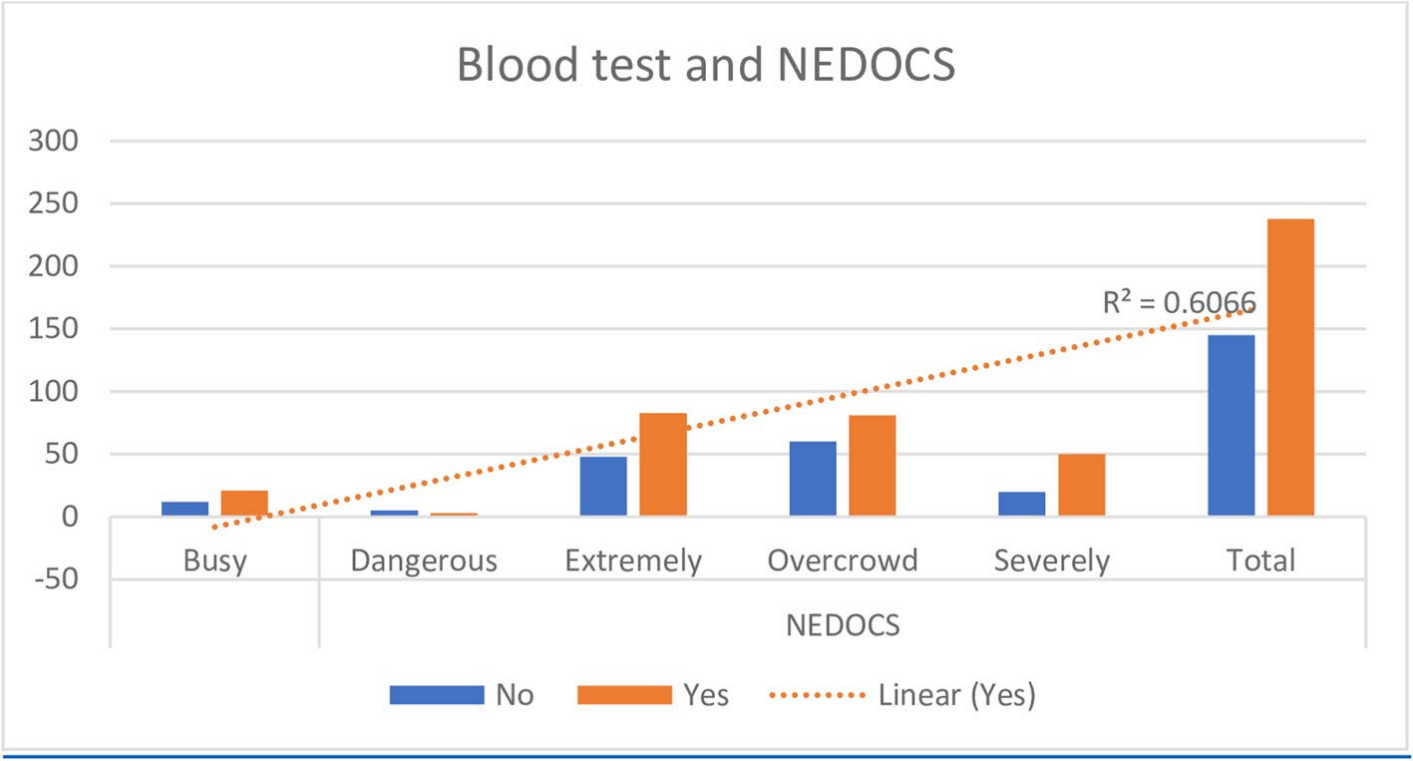
Correlation between blood-taking habits and the level of busyness in the department

Advancing age which was categorised, EDLOS and higher acuity were associated with increased blood testing (Table 1.). However, when triage category and ED length of stay were controlled, age had no statistically significant association with blood test performance (*p* = 0.119). While blood testing was not associated with how busy the department was according to the NEDOCS (*p* = 0.230), there was more tendency to perform a blood test during busy times, with extremely busy times (*n* = 130) having a 63% bleed rate, overcrowded (*n* = 141) 57% and severely overcrowded (*n* = 70) 71% bled (Fig. 2).

As anticipated, there was a statistically significant association between EDLOS and NEDOCS (*p* = 0.015) and statistically significant correlation between arrival-bleeding time and EDLOS (*P* < 0.001) but there was no correlation between time of day presented to ED and LOS (*p* = 0.930). Of the 238 participants who had blood tests, 71% (*n* = 169) were found to have stayed longer than the target 4 h as stipulated in the National Emergency Access Target [12] with an average of 8.5 h.

Test relevance to diagnosis and treatment among those who had blood test was assessed by the three study reviewers as described above. Though blood tests performance with the frequency of association distributed as; Yes = 338, No = 88, undetermined = 50 was statistically shown to be associated with diagnosis (*p* < 0.001, CI, -0.417 to -0.379) and treatment (*P* < 0.001, CI, -0.356 to -0.308) the overall low measure of interrater agreement for those who had blood tests with a Kappa of 0.598 and 0.299 respectively casts doubt on this association. Patients’ final destinations and chances of being tested were found to be associated (*p* < 0.001) with those patients admitted to hospital being tested more.

## Discussion

Demands for ED services have been increasing over the years [13–15]. Overall, the impact of the COVID − 19 pandemic has seen an increase in demand following an early phase of hospital avoidance [16–18]. Our study took place during multiple waves of the pandemic in which the ED was ‘extremely busy’ or worse (based upon the NEDOC score) for 91% of the time and was never “subdued”.

Given the stretched resources of the department [18] and particularly the nursing staff’s time demand, it is worthwhile revisiting and re-evaluating our ED processes. Routine ED blood tests performance has both its critics [5, 6, 19] and complementors [4, 20] and it is time to critically evaluate this practice. Emergency departments in Australia are guided by ACEM and RCPA joint guideline on pathology testing in the ED [3]. Cost analysis of performing these tests is beyond the scope of this study but the literature indicates cost benefits in relation to using alternative tests like point of care (POC) tests and saving time [21, 22].

Among the routine bloods test found to have been performed, FBE and UEC were the most common tests. It is possible that a large proportion of these tests can be avoided [5, 23]. An alternative to routine pathology tests is the use of POC testing. Studies have shown this to be promising especially with senior clinicians’ engagement and consideration [22, 24–27].

Blood test performance was associated with age, level of acuity, and increased EDLOS, while age was an independent predictor of EDLOS. Other studies have shown an association between investigations in the older ED patient and length of stay in the ED [28–30] raising the question of whether there is an element of “over testing” older patients as demonstrated in another study [31].

The relevance of performing routine blood tests to aid diagnosis and treatment decision-making has been questioned for a long time [2]. At triage where detailed history taking and examination cannot be guaranteed, performing these tests can raise questions about appropriateness. This is of lesser importance if testing is part of established and validated clinical guidelines [3]. We were unable to ascertain the relevance of testing in this study, given the interrater reliability.

Australia has a well-structured general practitioner (GP) service that follow-up their patients and conduct routine investigations, therefore, majority of older patients visiting EDs could be managed by their GP [32] and care coordination between EDs and GPs could be improved.

This study has important limitations as it was a single centre study and hence there are limits on generalisability to other populations, the study design being retrospective observational which usually has inferior level of evidence compared to prospective studies. There is sufficient evidence of association between blood test with diagnosis and treatment but acknowledge that the low measure of interrater agreement resulting from methodological flaws due to non-blinding of the reviewers and therefore, requires consideration when interpreting. Also, the inter-rater reliability suffered from biases on the possibility of lack of comprehensive documentation on blood works influence on diagnosis and treatment decision making and also due to the fact that, it was tested only for those who had blood tests.

The study lacked adequate statistical power to establish causality for certain observed associations. This limitation is common in emergency medicine research, where measures of patient flow and outcomes are intricately linked to numerous shared variables, thus complicating the determination of causal relationships.

## Conclusion

Blood test performance in the department among non-time critical patients based on allocated triage category was found to be high. Our study has shown evidence of the emergency department being constantly busy or very busy as per the NEDOC scale leading to a higher tendency of blood testing. There has also been positive association between blood test performance and the length of stay in the department.

Review of institutional practice on patient’s blood testing criterion, utilisation of POCT and targeted strategies to minimise routine blood tests, particularly in the older ED patients (with qualifying criteria like exclusion of category 3’s in the potentially life-threatening situations range of the category) may help decongest the department and improve patient satisfaction.

## Data Availability

Data will be provided where reasonable request is made.
